# Recent Veterinary Literature on Surgery1Presented to the Minnesota State Veterinary Medical Association, July 11, 1900.

**Published:** 1901-01

**Authors:** M. H. Reynolds

**Affiliations:** University of Minnesota, Experiment Station, and State Board of Health


### RECENT VETERINARY LITERATURE ON SURGERY.[1]

By M. H. Reynolds,
UNIVERSITY OF MINNESOTA, EXPERIMENT STATION, AND STATE BOARD OF HEALTH.

The following summary of an article by Huber, in the *Journal of Physiology*, February, 1900, confirms modern teaching on the subject of regeneration of nerve-fibres, and is especially interesting perhaps to those who are doing frequent neurectomies: "1. The motor and sensory nerve-endings of voluntary muscle degenerate after severance of the muscular nerves. The motor nerve-endings and the extreme distal portion of the motor nerves degenerate earlier than do the sensory nerve-endings. 2. The motor and sensory nerve-endings in voluntary muscle may, under suitable conditions, regenerate completely. The motor nerve-endings regenerate more quickly than the sensory nerve-endings. 3. These experiments, it seems to me, show clearly that the regeneration of a degenerated portion of a peripheral nerve is brought about by the down growth of the axis-cylinder of the central, undegenerated portion of the nerve-fibre."

[1] Presented to the Minnesota State Veterinary Medical Association, July 11, 1900.

The *Veterinarian* (London), May, contains an interesting article on perforating wounds of the neck, with rupture of the pneumogastric and sympathetic nerves. This case is especially interesting, as the reported symptoms were referable mainly to the alimentary canal. Certain secondary symptoms appearing later involved the respiration and heart-action.

In the same number of this journal is reported a study by Hocke of the way in which the lesions of actinomyces spread. These conclusions may be briefly but rather imperfectly summarized as follows: That there is a constant struggle between the leucocyte and the invading organism. In some cases the leucocyte seems to incorporate within itself filaments of the mycelium and increase greatly in size. It may be so large that it is unable to re-enter the blood-stream. If the leucocyte succeeds in destroying the parasite it returns to normal dimensions and conditions, but in many cases the leucocyte is not victorious and becomes the victim of the parasite it attempts to destroy. Such leucocyte cells then die, and the victorious actinomyces send out branches and continue to develop, forming new colonies. The leucocytes, or white blood-cells, in this way appear as active agents in spreading the infection. This explains the rather slow invasion of healthy tissue immediately bordering on the seat of original inoculation.

Dr. Doyen, Paris, in a French scientific journal, discusses his ideas and methods of teaching surgery by the use of the cinematograph (practically the French name for kinetoscope). These moving pictures reproduce the operation as actually performed. In this way a very large number of students are permitted to follow the operation very closely. An actual operation can be performed and then afterward reviewed and fully discussed by the aid of this instrument. As a rule, comparatively few students are able to take actual part in critical operations or even to watch closely the various proceedings. The first illustration of the use of this instrument in teaching surgery was made before the British Medical Association in 1898. In a French medical college a course in the study of surgery has been established to be illustrated by means of this instrument. Not only can students witness the operation in this way, but the operation can be as perfectly reproduced thousands of miles away, and it is possible that American surgeons can save considerable of their time by studying the operating methods in this way at home.

A German surgeon has developed a device which enables the interior of the stomach to be photographed for the purpose of diagnosis. The camera is exceedingly minute and intended to be swallowed by the patient. As soon as it reaches the stomach the walls are illuminated by means of a small electric lamp. The narrow film, twenty inches long, is connected with a cord, the cord to be pulled by the operator. Conducting wires are of course swallowed with the camera and cord.

The June number of the JOURNAL OF COMPARATIVE MEDICINE AND VETERINARY ARCHIVES published a practical article by Dr. J. Payne Lowe, of New Jersey, detailing actual experiences with median neurectomies. It is scarcely necessary to call attention to the great importance of this kind of information. Dr. Lowe first gives in detail methods of confining the patient and performing the operation. There is given nothing new in either, but the details are quite carefully described, so that one not familiar with the operation could quite easily follow the various steps as described. (I would suggest in criticism that if the incision is made a trifle lower the nerve is more easily found ; in fact, at a somewhat lower point the nerve can in most subjects be clearly defined by sense of touch and the incision made directly upon it. I also prefer to hold the lips of the wound open by a simple retractor made from common rubber-bands, with a small pin hook at each end. This is self-retaining, no difference how much the horse struggles, and there is no blunt tenaculum or assistant's hand in the operator's way.)

Dr. Lowe urges a surgically clean operation, followed by cold-water irrigation if the weather permits; and with this treatment expects to have complete healing in about two weeks. In this article seven operations are reported, with the following results: In two cases of ring-bone, operations failed to give good results, there being ankylosis, and of course some mechanical lameness. One case of navicular disease was successful. Four cases of tendonitis were all successful. Dr. Lowe concludes with the suggestion that we should not perform this operation when it is unnecessary. A very good suggestion.

In the same number of this JOURNAL Dr. W. T. Campbell reports upon protargol as a new treatment for fistula. This is described as a light yellowish powder, freely soluble in water, and a derivative of silver, but less irritating than the nitrate. Dr. Campbell reports two cases of fistula of the withers and one of quittor treated with this agent. I might suggest in criticism that the results obtained from two cases of fistula cannot be taken as evidence of much importance in estimating the value of a new treatment ; but, on the other hand, Dr. Campbell is to be commended for reporting his experience, and if members of this Association decide to do any experimenting along this line in the near future we should all be glad to learn their results at our next meeting.

Dr. L. A. Merillat, in the May number of the *American Veterinary Review*, makes some interesting statements concerning the use of chloroform upon horses. He notes that the time required for reaching surgical anæsthesia is usually stated as from fifteen to forty minutes. Moller gives seven minutes with 35 grammes of chloroform as his shortest record, and the average among one hundred and twenty-six patients varied from eighteen to twenty-two minutes. Commenting upon this, Merillat states that he has never failed to obtain profound anæsthesia in less than three minutes, and frequently in thirty seconds. After some discussion and comparison of slow and rapid methods he concludes that the dangers are scarcely worth mentioning if proper care is taken, and that laboring over a patient for twenty to forty minutes is at least unnecessary.

A case of collection in the maxillary sinuses of a cow is interesting chiefly on account of its rarity. Such a case is reported in the same number of the *Review*. I have never seen a case of this in cattle practice, and have wondered whether other members of the Association had been more fortunate.

Dr. W. Hunting has a very interesting article in the *Veterinary Journal* (English) for May, 1900, on shoulder-lameness. He first discusses the indefiniteness of the term. The chief interest and value of his article lie in the thoroughness with which he attempts to upset most of our previous teaching and opinions concerning this peculiar trouble. He cites the various symptoms that have usually been considered diagnostic of shoulder-lameness, and in nearly every case points to some other form of lameness which produces the same symptoms. For instance, concerning the good old symptom, circumduction of the leg, he shows that some forms of lameness having their seat in the knee or foot occasionally show this symptom. Abduction is discussed as quite common in foot-troubles and even in injuries of the pectoral muscles. Motion and manipulation are discussed as deceptive. The author admits the use of these tests, at the same time confessing that he rarely succeeds in getting any satisfactory information by them. Position of the limb at rest is considered very helpful, but, on the other hand, it utterly fails in many cases to give any useful information.

I presume that most members of this Association have read the

*American Veterinary Review* for June, and have thought over Dr. Hannawalt's article on chromic acid. To those who have not I would simply report that the doctor recommends this agent quite strongly, especially as a caustic in the treatment of such cases as fistula and other chronic sores. He gives some caution concerning the use of the drug, particularly as to severe action upon the operator's hands. He recommends the cautious use of a 0.2 per cent. solution or 1 : 500.

In the same issue of this journal there is quite an article on paraldehyde in veterinary practice, taken from a foreign journal. This drug is ranked by its chemical properties as standing between alcohol and ether. Diluted with water or glycerin it has a peculiar odor, antiseptic and hypnotic action. The drug is described as not satisfactory for producing deep sleep, but as acting peculiarly on the sensitive nerves, thirty grammes, approximately an ounce, given in capsules, permitting painless performance of severe operations.

The following notes concerning local anæsthetics are taken mainly from the *Journal of Comparative Pathology and Therapeutics.* Cocaine dissolves rapidly in either hot or cold water, changed chemically by boiling, and rendered ineffective; should usually be made fresh, but when 2 per cent. of carbolic acid is added it keeps fairly well; immediate local effect occurs from two to five minutes, about fifteen minutes required for anæsthetizing the larger nerves, like the digital or plantar; anæsthesia lasts from twenty to thirty minutes; extent of area anæsthetized depends upon the way the circulation is controlled and upon the tissue into which it is injected. Eucaine used alone is slower in action and does not produce as complete anæsthesia, but the effect when once produced lasts much longer; has the advantage over cocaine in that it may be boiled for sterilization without decomposition; it has the further advantage of being considerably cheaper than cocaine; a mixture of eucaine and cocaine in some respects preferable to either alone, especially as effect lasts longer. Holocaine has something of an antiseptic action, and like eucaine is not decomposed on boiling; it is not suitable for general use, as it is considered somewhat more dangerous than cocaine; used chiefly for the eyes; not very commonly used. Orthoform, more recently introduced, seems to be especially valuable for large surfaces of wounds and mucous membranes; non-poisonous; can be used over large surfaces very safely. The writer has recently commenced the use of chloretone, but is not as yet prepared to express any opinion as to its value.

Dr. E. Merillat gives some interesting surgical "nevers" in the June *Review*. Several of these appeal to me quite strongly, for instance: Never operate without exhausting every possible means of making a correct diagnosis. Never operate without familiarizing yourself with the operation to be performed. Never foster dexterity (and speed) at the expense of judicious technique. Never perform an operation requiring delicate manipulation without the aid of an anæsthetic. To these I would like to add: Never commence an operation until you are ready. I occasionally violate this one myself, but nevertheless I dislike to see a surgeon commence an operation, then chase off, first after one instrument and then another, stopping to hunt for bandages or learning for the first time that he has no cocaine or his hypodermatic needles are all lost or broken. And I would add one other to this list: Never pick or piece; make clean, free incisions as long as needed at the first stroke, and do not keep on picking and picking after the operation is practically finished and the horse should be released.

Members of this Association who are especially interested in practical disinfection should secure a copy of the Lomb Prize Essay on "Disinfection and Individual Prophylaxis against Infectious Diseases," by Dr. Sternberg. This essay was originally written in 1886, and soon became an accepted authority and guide for practical disinfection. The author has recently made a revision which includes newer antiseptics and newer methods, bringing the whole thing up to date. Copies may be secured by writing H. Lomb, Superintendent of Essay Department, American Public Health Association, Rochester, N. Y.

The fifth series of one hundred laparotomies is reported by Dr. Heinricius, quoted in the *St. Paul Medical Journal* for April. This series of one hundred laparotomies was followed by four deaths. At least two were inevitable, as shown by post-mortems. I bring this item before a veterinary association for two reasons: first, because we should all be interested in problems of comparative medicine; and, second, as calling attention to the wonderful strides that have been made in human surgery.

I have been somewhat interested in an article published by the *Medical Record*, April 7, 1900, on "Summary of Cases of Carcinoma and Sarcoma Treated by Cataphoric Sterilization." This process may be briefly described as the diffusion of nascent mercuric salts produced by electrolysis, somewhat similar to the process used by dentists in producing anæsthesia of a very sensitive tooth by means of cocaine and the electric current. Of thirty-seven cases reported ten are given as cured; uncertain, seven; failures, twenty-two. Many of these cases are, of course, necessarily hopeless. Members of the Association who are especially interested in this could doubtless secure for perusal a copy of the *Medical Record* from some neighboring physician, as it has a large circulation.

The *Veterinarian* (English), June, 1900, discusses under "Clinical Cases," by Professor Cadiot, translated by Dr. Dollar, among other things an operation for rupture of the perineum. I consider this an operation which could frequently be performed with complete success, and yet one which is very commonly neglected as hopeless or useless. Professor Cadiot reports an operation on a complete rupture of the perineum, but which did not involve the rectum. The length of time which had elapsed since the accident had occurred is not given, but it is mentioned that an operation had been attempted by another veterinarian which resulted in failure. It was probably, therefore, what we might call an old case. From the 19th to the 26th of October the patient was prepared by warm injections of antiseptic solution and had sodium sulphate internally. The surfaces involved in the operation were thoroughly washed and then disinfected. The skin and mucous membrane were divided throughout the entire length of the tear; suitable surfaces were secured for healing by removing a strip of mucous membrane extending to the skin edge. The surfaces were then brought together by a double suture, which is carefully described in the article. Subsequent treatment is described until November 3d, at which time union was complete, except at the upper part of the wound, where a few of the deep sutures had given away. The upper margin of the wound was again prepared and sutured, the patient being discharged as cured on December 6th. I have operated on several of these cases, and all except my first case have been successful. I do not employ the double suture as described by Professor Cadiot, but merely the old-fashioned quill suture, deeply inserted.

